# Comparison of Glomerular Filtration Rate Estimation from Serum Creatinine and Cystatin C in HNF1A-MODY and Other Types of Diabetes

**DOI:** 10.1155/2015/183094

**Published:** 2015-08-10

**Authors:** Magdalena Szopa, Maria Kapusta, Bartlomiej Matejko, Tomasz Klupa, Teresa Koblik, Beata Kiec-Wilk, Maciej Borowiec, Maciej T. Malecki

**Affiliations:** ^1^Department of Metabolic Diseases, Jagiellonian University Medical College, 15 Kopernika Street, 31-501 Krakow, Poland; ^2^University Hospital, Krakow, Poland; ^3^Department of Diagnostics, Jagiellonian University Medical College, Krakow, Poland; ^4^Department of Clinical Genetics, Medical University of Lodz, Lodz, Poland

## Abstract

*Introduction.* We previously showed that in HNF1A-MODY the cystatin C-based glomerular filtration rate (GFR) estimate is higher than the creatinine-based estimate. Currently, we aimed to replicate this finding and verify its clinical significance.* Methods.* The study included 72 patients with HNF1A-MODY, 72 with GCK-MODY, 53 with type 1 diabetes (T1DM), 70 with type 2 diabetes (T2DM), and 65 controls. Serum creatinine and cystatin C levels were measured. GFR was calculated from creatinine and cystatin C using the CKD-EPI creatinine equation (eGRF-cr) and CKD-EPI cystatin C equation (eGFR-cys), respectively.* Results.* Cystatin C levels were lower (*p* < 0.001) in the control (0.70 ± 0.13 mg/L), HNF1A (0.75 ± 0.21), and GCK (0.72 ± 0.16 mg/L) groups in comparison to those with either T1DM (0.87 ± 0.15 mg/L) or T2DM (0.9 ± 0.23 mg/L). Moreover, eGFR-cys was higher than eGRF-cr in HNF1A-MODY, GCK-MODY, and the controls (*p* = 0.004; *p* = 0.003; *p* < 0.0001). This corresponded to 8.9 mL/min/1.73 m^2^, 9.7 mL/min/1.73 m^2^, and 16.9 mL/min/1.73 m^2^ of difference. Additionally, T1DM patients had higher eGFR-cr than eGFR-cys (11.6 mL/min/1.73 m^2^; *p* = 0.0004); no difference occurred in T2DM (*p* = 0.91).* Conclusions.* We confirmed that eGFR-cys values in HNF1A-MODY patients are higher compared to eGFR-cr. Some other differences were also described in diabetic groups. However, none of them appears to be clinically relevant.

## 1. Introduction

Diabetic kidney disease (DKD) is the leading cause of end-stage renal disease (ESRD) and is associated with an increased risk of cardiovascular mortality [1, 2]. In spite of substantial progress in treatment and monitoring methods, the risk of ESRD remains high in both type 1 (T1DM) and type 2 diabetes (T2DM) [3, 4]. Monitoring of DKD occurrence and progression is considered crucial for prevention of kidney damage. Over recent decades, clinical guidelines recommended measuring albumin excretion rate in order to monitor progress of DKD [5]. However, recent scientific data suggest that the loss of glomerular filtration rate (GFR), rather than albuminuria, should be the main outcome in diabetic kidney research and clinical practice [6]. In everyday manner, GFR is measured using one of two estimates. The first is based on serum creatinine level while the second estimate is built on serum cystatin C level [7, 8]. The Chronic Kidney Disease Epidemiology Collaboration (CKD-EPI) has provided a formula for each estimation. Cystatin C is a 13-kDa cysteine proteinase inhibitor and is produced by all nucleated cells at a constant rate. In healthy subjects, cystatin C is freely filtered by the renal glomeruli and almost entirely reabsorbed in the proximal tubule like other low molecular weight proteins [9]. The cystatin C GFR estimate has recently been recommended with increasing frequency as having important advantages over its alternatives [8].

We previously postulated that cystatin C level might also be a biomarker for HNF1A-MODY, a monogenic form of diabetes, as concomitant kidney phenotypes were described in this form of disease [10]. Our hypothesis was excluded by examining three different HNF1A-MODY cohorts, one from Poland and two from the UK. Interestingly, in all examined HNF1A-MODY groups, we consistently observed that the cystatin C-based GFR (2008 equation) estimate was higher than the creatinine-based one. The difference between the estimates reached 25 mL/min/1.73 m^2^ in one cohort. Such a difference may be of clinical importance and lead to inappropriate clinical decisions. This previous finding required further confirmation. Moreover, since then, a new equation has been recommended for cystatin C eGFR calculation [8].

In the current study, we aimed to replicate our previous finding that in HNF1A-MODY patients estimation of GFR from serum cystatin C is higher than from serum creatinine and to verify whether this difference is clinically relevant.

## 2. Material and Methods 

The study included 72 patients with HNF1A-MODY, 72 with GCK-MODY, 53 with T1DM, and 70 with T2DM as well as 65 control subjects. All patients received the medical care at the Department of Metabolic Diseases, University Hospital in Krakow, a tertiary referral center for diabetes care in southeastern Poland.

The individuals were considered as diagnosed with diabetes if at the study entry they were on hypoglycemic treatment or met the diagnostic criteria based on fasting glucose level measurements. The subjects were white Caucasians, the residents of southeastern Poland. All examined MODY patients had a molecular diagnosis established during research activities performed at the Jagiellonian University Medical College [11, 12]. The pathogenicity of mutations was determined based on earlier reports, DNA sequence difference molecular character, and segregation within the families. Subjects with T1DM were identified if at diagnosis they had typical clinical symptoms, an insulin therapy requirement from the beginning of the disease, and diabetes diagnosed below 30 years of age. We also included patients with a clinical diagnosis of T2DM who at least 2 years after diagnosis remained without insulin therapy. The control group consisted of apparently healthy subjects with normal fasting glucose levels. No individual with known thyroid gland function abnormality or on steroid therapy was included into this study. Subjects received a standard questionnaire and underwent a basic physical examination. For all study individuals, we collected data on their clinical characteristics. None of the individuals from the GCK-MODY, T2DM, T1DM, and control group was evaluated in our previous report. Twenty-two patients from the HNF1A-MODY group were previously included; however, they were currently invited for a new examination and sample draw. We excluded all individuals with active infections, neoplasms, severe chronic diseases of the respiratory tract, kidney, or liver as well as pregnant women. This study was performed according to the Helsinki Declaration and it was approved by the Bioethical Committee of the Jagiellonian University.

We performed clinical laboratory analyses, such as HbA1c level, fasting glucose, lipids, CRP, creatinine level, and the urinary albumin-to-creatinine ratio (ACR). The previously described methods were used [11, 12]. Serum cystatin C was measured using an immunoturbidimetric method (APTEC Diagnostics nv, Belgium) on the Maxmat PLII clinical chemistry analyzer (Maxmat S. A., Montpellier, France) calibrated against the international certified reference material ERM-DA471/IFCC. Estimated GFR was calculated from serum creatinine and cystatin C level using the CKD-EPI formula [7, 8].

Statistical analysis was performed to determine the difference between two (*t*-student) and several groups (ANOVA with post hoc tests). If necessary, nonparametric tests were utilized as equivalents. Predictive multivariate linear regression analysis was used to assess if differences between both eGFRs (counted from cystatin C and creatinine) changed in diagnosis groups after adjustment for gender, age, BMI, glucose level, CRP concentration, HDL level, and total cholesterol level. Statistical analyses were performed using STATISTICA ver. 10.0 and R ver. 3.1.1 software.

## 3. Results

The study groups' characteristics are shown in Table 1. Distribution variations among diabetic groups for age, BMI, and diabetes duration were in line with the way the groups were defined. We identified 8 patients with chronic kidney disease (CKD) defined as CKD-EPI creatinine equation (eGRF-cr) <60 mL/min/1.73 m^2^; there were 5 such individuals in the T2DM group, 2 in HNF1A-MODY group, and 1 in T1DM group.

Differences in cystatin C level were identified between study groups (*p* < 0.001). In the post hoc analysis (Tukey test) cystatin C levels were significantly lower (*p* < 0.0016) in the control (0.70 ± 0.13 mg/L), HNF1A-MODY (0.75 ± 0.21 mg/L), and GCK-MODY (0.2 ± 0.16 mg/L) groups in comparison to those with either T1DM (0.87 ± 0.15 mg/L) or T2DM (0.9 ± 0.23 mg/L).

We observed that eGFR-cys was higher than eGRF-cr in three groups: HNF1A-MODY, GCK-MODY, and controls (*p* = 0.004; *p* = 0.003; *p* < 0.0001) as shown in Table 1. This corresponded to rates of 8.9, 9.7, and 16.9 mL/min/1.73 m^2^, respectively. Contrarily, in T1DM patients, eGFR-cr was higher than eGFR-cys (11.6 mL/min/1.73 m^2^; *p* = 0.0004). No significant difference between both estimates was observed in T2DM (*p* = 0.91).

We further analyzed if differences between both GFRs remained significant in the multivariate regression model. It was revealed that differences between the GFR estimates within groups remained almost unchanged and, thus, unrelated to main clinical features.

Additionally, we determined the median eGFR-cr in every group. The range of the medians varied between 115 mL/min/1.73 m^2^ in T1DM and 92 mL/min/1.73 m^2^ in T2DM. We then calculated differences between eGFR-cr and GFR-cys in subgroups below and above the eGFR-cr median in every diagnosis group. For the “above median” analysis, the range of eGFR-cr and GFR-cys differences varied from 18.2 mL/min/1.73 m^2^ in T1DM to −0.5 mL/min/1.73 m^2^ in T2DM. In the HNF1A-MODY group it reached −2.4 ± 19.0 (*p* = 0.22), whereas, for the “below median” comparisons, the differences ranged from −1.5 mL/min/1.73 m^2^ in T2DM up to −22.1 mL/min/1.73 m^2^ in the controls. In the HNF1A-MODY, GFR-cys was higher than GFR-cr by −14.6 ± 16.6 (*p* < 0.0001).

We also performed all analyses after excluding 22 HNF1A-MODY patients whose earlier specimens were examined in our previous report. This exclusion did not substantially change the study results (data not shown).

## 4. Discussion 

In this study, we confirmed that eGFR-cys was higher than eGRF-cr in HNF1A-MODY. Moreover, this difference was found to be unrelated to the main clinical features of the examined patients. This is in line with our previous results from three cohorts: one from Poland and two from the UK [10]. Nevertheless, the magnitude of the difference between both estimates does not appear to have a clinical significance.

Impaired GFR is, together with increased albumin excretion rate, a sign of diabetic kidney damage. GFR is in clinical practice estimated, rather than measured, using serum creatinine level as the most common approach. However, the creatinine level is influenced by a number of clinical factors, such as age, muscle mass, sex, and race [13]. Given these limitations, serum cystatin C has been proposed as an alternative GFR marker [9]. Cystatin C is less affected by features influencing creatinine level; moreover, in the general population it associates more strongly with all cause and cardiovascular mortality than does serum creatinine [14]. A significant association was found between cardiovascular mortality and low eGFRcys, but not eGFRcr, among diabetic patients from the large US NHANES registry [15]. The clinical utility of cystatin-based eGFR was also demonstrated in another clinical study in which only the cystatin C-based chronic kidney disease definition was an independent risk predictor for cardiovascular events [16]. However, factors such as BMI, diabetes, and inflammation may impact cystatin C levels to some degree independently of kidney function [9]. In general, results obtained for eGFR-cr highly correlate with those calculated from eGFR-cys [17]. However, it was suggested that, in persons with diabetes, cystatin C-based estimate use would result in a higher prevalence of reduced renal function than would estimate based on serum creatinine [15]. Moreover, reclassification from preserved kidney function defined based on creatinine level to reduced kidney function using cystatin C occurred more often among persons with diabetes than among those without [15].

There were some earlier reports comparing both estimates in common forms of diabetes. It was shown that in T1DM GFR estimated from cystatin C reflects normal and elevated renal function better than its creatinine alternative even during hyperglycemia [18]. It was also suggested that the addition of cystatin C to creatinine to estimate GFR may improve identification of the causes and consequences of GFR loss in T1DM [19]. On the other hand, a comparison of GFR estimated from creatinine and cystatin C with measured GFR in a large group of participants consisting exclusively of T2DM with a broad range of renal function showed no evidence of cystatin C-based approach superiority [20]. However, in T2DM patients with poor glycemic control, the eGFR-cys was shown to be less biased and more accurate than the creatinine-based formula [21]. So far, to our knowledge, there has been no study evaluating cys-GFR in any rare type of diabetes, apart from our earlier report [10]. Interestingly, the T1DM group in the current study was the only one with a higher eGFR-cr than eGFR-cys. A satisfactorily comprehensive interpretation for this observation is not available and replication of this finding is necessary. A possible explanation could point to differences in glycemic control between different diabetes types in our study. Of note, it was earlier shown that eGFR-cr tends to overestimate glomerular filtration as assessed by the reference inulin method in T1DM under euglycemic conditions. During hyperglycemic clamp, however, creatinine-based calculations underestimated the inulin-based assessed GFR [18]. The impact of glucose values characterizing the current study T1DM cohort on eGFR-cr in everyday setting is unknown.

A cause of detected differences in HNF1A-MODY is not clear. One may hypothesize that they are related to some abnormalities in cystatin C synthesis or kidney reabsorption. For example, HNF1A directly regulates the expression of the chloride-proton exchanger ClC-5, which is essential for the endocytic activity of the proximal tubule cells and the tubular clearance of proteins filtered in the glomeruli [22]. It was also shown in an animal model that structural changes, such as diffuse glomerular nodules, were present in pigs carrying a dominant-negative P291fsinsC mutation of the HNF1A gene [23].

Our results should be interpreted within the limitation of the study. First, we did not have a reliable gold standard for GFR assessment. Performing inulin or iothalamate clearance tests implies invasive and tedious procedures that lied outside of the current research. Moreover, our study groups were small, and we should acknowledge that much larger cohorts were examined in earlier studies for the control population, T1DM and T2DM; the size of these groups limited statistical power of the analysis. Additionally, our study groups differed in terms of some clinical characteristics; these differences were definition linked with the clinical picture of the examined forms of diabetes. Also, the majority of subjects included in this research had an eGFR above 60 mL/min/1.73 m^2^, which makes our conclusions inapplicable to advanced stages of renal disease. Finally, we did not perform a direct assessment of any tubular defect in HNF1A-MODY and other study groups [24]; instead, our investigations were limited to its putative indirect effect on serum cystatin C or creatinine level. In spite of these shortcomings, taking into account the replication character of our HNF1A-MODY finding, we consider our main conclusion to be well justified.

In summary, we confirmed that GFR values estimated from serum cystatin C levels in HNF1A-MODY patients are higher compared to eGFR from creatinine. Some other differences were also described in remaining diabetic groups. However, none of them appears to be clinically relevant.

## Figures and Tables

**Table 1:** Clinical characteristics of patients and controls without diabetes.

Clinical characteristics	HNF1A	GCK	T1DM	T2DM	Controls	*P* value^*∗*^
Gender F *N* (%)	26 (36.1)	29 (40.3)	21 (55.7)	39 (55.7)	23 (35.4)	0.0990
Age at the examination (years)	40.28 ± 14.77	36.83 ± 14.65	31.72 ± 11.71	58.96 ± 10.25	38.02 ± 11.70	<0.0001
Age at diagnosis (years)	24.95 ± 10.96	26.19 ± 13.09	18.58 ± 10.97	52.16 ± 10.34	—	<0.0001
Diabetes duration (years)	16.7 ± 11.02	10.58 ± 8.15	12.81 ± 9.45	6.8 ± 6.41	—	<0.0001
Body mass index (kg/m^2^)	24.05 ± 4.00	23.64 ± 4.40	24.43 ± 3.20	30.48 ± 4.76	23.91 ± 2.93	<0.0001
Fasting glucose (mmol/L)	6.91 ± 2.68	6.82 ± 1.21	7.92 ± 3.18	7.52 ± 1.94	5.12 ± 0.53	<0.0001
HbA1c (%)	6.83 ± 1.41	6.33 ± 0.67	7.41 ± 1.30	7.04 ± 1.33	—	<0.0001
C-peptide (ng/mL)	1.33 ± 0.66	1.31 ± 0.98	0.20 ± 0.25	2.41 ± 1.23	1.55 ± 0.66	<0.0001
Total cholesterol (mmol/L)	4.79 ± 0.97	5.05 ± 1.09	4.41 ± 0.76	4.61 ± 0.97	5.04 ± 0.81	0.0005
LDL-C (mmol/L)	2.64 ± 0.86	2.97 ± 0.85	2.44 ± 0.72	2.56 ± 0.84	3.04 ± 0.76	<0.0001
HDL-C (mmol/L)	1.59 ± 0.42	1.62 ± 0.42	1.63 ± 0.43	1.23 ± 0.37	1.53 ± 0.41	<0.0001
Triglycerides (mmol/L)	1.23 ± 0.92	0.93 ± 0.36	0.85 ± 0.36	1.82 ± 1.02	1.01 ± 0.49	<0.0001
Creatinine (mmol/L)	70.39 ± 15.26	70.46 ± 14.33	68.63 ± 14.77	75.34 ± 17.61	74.4 ± 13.12	0.0646
CRP (mg/L)	0.71 ± 1.16	1.69 ± 2.85	1.53 ± 1.47	3.2 ± 5.06	0.95 ± 0.89	<0.0001
Cystatin C (mg/L)	0.75 ± 0.21	0.72 ± 0.16	0.87 ± 0.15	0.9 ± 0.23	0.70 ± 0.13	<0.0001
Urine albumin/creatine ratio (*μ*g/mg)	6.38 ± 29.62	2.72 ± 13.06	3.66 ± 9.27	7.38 ± 17.85	—	0.0439
eGFR-cys (mL/min/1.73 m^2^)	110.94 ± 21.57	113.73 ± 18.49	100.02 ± 17.55	89.64 ± 21.64	116.24 ± 15.56	<0.0001
eGFR-cr (mL/min/1.73 m^2^)	102.86 ± 19.32	105.14 ± 18.13	111.48 ± 15.8	88.66 ± 16.99	99.45 ± 14.35	<0.0001
eGRF-cys - eGFR-cr (ml/min/1.73 m^2^)	−8.9 ± 18.7	−9.7 ± 17.0	11.6 ± 21.2	−1.00 ± 16.3	−16.9 ± 15.0	<0.0001

Data are presented as mean and standard deviation (SD). For categorical variable numbers and percentage were used.

^*∗*^ *P* value derived from one-way analysis of variance (ANOVA) or Kruskal-Wallis test to detect a significant difference in the variable levels among study groups.
